# Aggressions on Social Networks: What Are the Implications for Healthcare Providers? An Exploratory Research

**DOI:** 10.3390/healthcare9070811

**Published:** 2021-06-28

**Authors:** Micaela La Regina, Arianna Mancini, Francesco Falli, Vittorio Fineschi, Nicola Ramacciati, Paola Frati, Riccardo Tartaglia

**Affiliations:** 1 SS Risk Management, ASL5 Liguria, 19124 La Spezia, Italy; micaela.laregina@asl5.liguria.it; 2 UOC Internal Medicine 4, Azienda Ospedaliero-Universitaria Pisana, 56126 Pisa, Italy; arianna.mancini409@gmail.com; 3 SS Professioni Sanitarie, ASL 5 Liguria, 19124 La Spezia, Italy; francesco.falli@asl5.liguria.it; 4 Department of Anatomical, Histological, Forensic and Orthopaedic Sciences, Sapienza University of Rome, Viale Regina Elena 336, 00161 Roma, Italy; paola.frati@uniroma1.it; 5 IRCSS Neuromed Mediterranean Neurological Institute, 86077 Pozzilli, Italy; 6 Settore Formazione e Qualità, Azienda Ospedaliera di Perugia, 06124 Perugia, Italy; nicola.ramacciati@ospedale.perugia.it; 7 Department Engineering Science, “G. Marconi” University, 00193 Roma, Italy; ri.tartaglia@unimarconi.it

**Keywords:** aggressions, healthcare providers, social networks, cyberbullying, law

## Abstract

Incidents of violence by healthcare users against staff have been considered as sentinel events. New forms of aggression, i.e., cyberbullying, have emerged with the advent of social networks. Medical literature includes some reports about workplace cyberbullying on nurses and young doctors by colleagues/supervisors, but not by users. To investigate cyberbullying on healthcare providers via social networks, we carried out an exploratory quali-quantitative study, researching and analyzing posts and comments relating to a local Health Trust (ASL5) in Italy, published from 2013 until May 2020 on healthcare worker aggressions on social networks on every local community’s Facebook page. We developed a thematic matrix through an analysis of the most recurring meaning categories (framework method). We collected 217 texts (25 posts and 192 comments): 26% positive and 74% negative. Positive posts were shared about ten times more than negative ones. Negative comments received about double the “Likes” than the positive ones. Analysis highlighted three main meaning categories: 1. lack of adequate and functional structures; 2. negative point of view (POV) towards some departments; 3. positive POV towards others. No significant differences were observed between the various categories of healthcare workers (HCW). Geriatric, medical wards and emergency department were the most frequent targets of negative comments. All the texts referred to first-line operators except for one. Online violence against HCW is a real, largely unknown, problem that needs immediate and concrete attention for its potentially disastrous consequences. Compared to traditional face-to-face bullying, it can be more dangerous as it is contagious and diffusive, without spatial, temporal or personal boundaries.

## 1. Introduction

In 2007 the Italian Health Ministry promoted a safety recommendation to prevent violence against healthcare providers, defining “violence” as “any physical assault, threatening behavior or verbal abuse that occurs in the workplace” [1]. The applications of social media to medicine have recently gained a lot of attention as social media enables the fast sharing of information [2]. However, the advent of social networks has led to the development of new forms of aggression, due to the emergence of “cyberbullying” and “keyboard warriors”—web users who write aggressively, offending, discrediting and threatening other users with sometimes disastrous consequences. Typically, cyberbullying is classified as an intentional, aggressive act or acts over a period of time to inflict harm on the victim by utilizing various electronic forms of expression [3]. In national and international law, hate speech refers to any kind of communication in speech, writing or behaviour that attacks or uses pejorative or discriminatory language with reference to a person or a group on the basis of who they are; in other words, based on their religion, ethnicity, nationality, race, colour, descent, gender or other identity factor. Trolling is a form of bullying that takes place in an online community to provoke a reaction, or simply for someone’s personal amusement [4]. Cyberstalking is a form of harassment that uses electronic communications to stalk a victim [5].

Currently in Italy, as in other European countries and in the USA, there is a law aimed at combating the phenomenon and protecting the victims [6]. According to data from the US Bureau of Labor Statistics, the incidence of violent events against healthcare workers increased by 67%, from 6.4 per 10,000 full-time workers in 2011 to 10.7 per 10,000 in 2018 [7]. At the European level, the EU has a role of coordinating and supporting the national initiatives of the member states and promoting directives on victims’ rights [8]. A survey of 226 nurses in Korea interviewed using a self-reported questionnaire showed that the explanatory power of the nursing organizational culture for face-to-face bullying was 6.3%, with relationship-oriented culture and hierarchy-oriented culture being the main factors influencing face-to-face bullying. The explanatory power of nursing organizational culture for cyberbullying was 4.3%, and relationship-oriented culture was one of the main factors influencing cyberbullying [9]. An online survey that was distributed to 1996 first-year trainee doctors (who had more than six months of training) and second-year trainee doctors showed interesting data [10]. Out of a sample of 73 respondents, 46.2% had been victims of at least one act of cyberbullying, with fellow trainees reported as the main perpetrators (35.6% = 26 respondents). In addition to trainees, 26.0% (19) of respondents cited consultants as perpetrators, 19.2% (14) attributed it to managers, 13.7% (10) could not specify further, 4.1% (3) reported nurses and 1.4% (1) cited patients or relatives as perpetrators [10]. Bullying is usually carried out by superiors, but other times bullying occurs between colleagues. This phenomenon is known as “horizontal bullying” and is a major problem, especially in the nursing profession [11].

A recent study showed how the COVID-19 pandemic has potentially made things worse. Indeed, a pre-pandemic survey conducted in 2019 showed that 23% of 464 US doctors said they had been personally attacked on social media [7]. Similarly, the prevalence of cyberbullying in the workplace has been estimated at 8% [12].

Currently, in the literature there are some reports on the use of social networks to measure patient-perceived quality of hospitals [13,14,15,16,17,18,19,20,21] and a few reports of cyberbullying in the healthcare workplace on nurses and young doctors by colleagues and supervisors [22], as well as healthcare worker aggressions on social networks [23], but not by healthcare users. We could not retrieve any work on this topic through a careful search on Medline, Embase and Scopus, unless utilizing anecdotal reports from seminars and meetings (Ramacciati N., personal unpublished data).

The present research aimed to explore the existence of cyberbullying on healthcare providers via social networks, using Facebook as it is the most popular community in our area.

## 2. Materials and Methods

**Figure 1:**
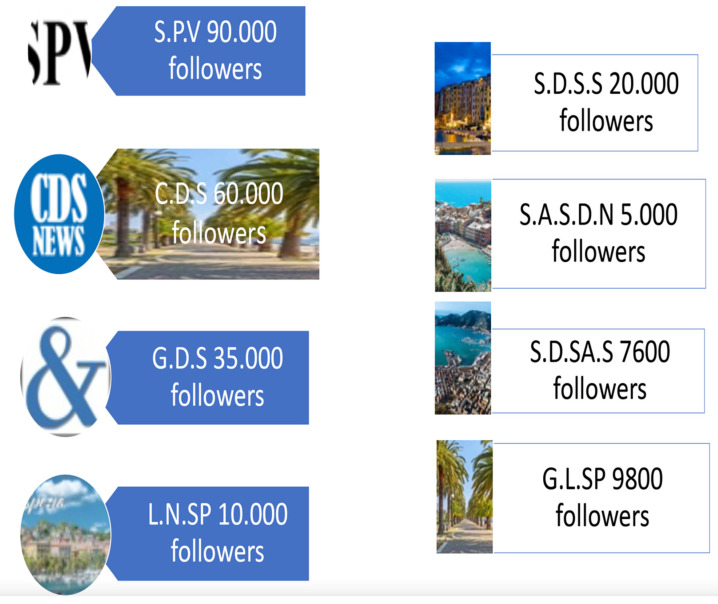
Facebook pages and groups analyzed in this research.

We carried out a quali-quantitative study, researching and analyzing posts and comments related to local Health Trust (ASL5 in Liguria, Italy), published from 2013 until May 2020 on four of the largest local Facebook pages (190,000 followers), on four main local Facebook groups (41,000 followers) and on one website of hospital reviews by patients (Figure 1). All pages are related to the city of La Spezia.

To select our population, we searched the mentioned Facebook pages/groups using the keywords “doctor,” “nurse,” “assault” and “hospital” to collect only posts referring to the NHS. In this case, posts not referring to a sanitary context were excluded (e.g., female assault, violence against other workers). Later, starting from the selected posts, we manually conducted a descriptive analysis of every comment using a reasoned choice sampling. We classified every comment in a binary way (negative or not negative). The classification was made using the following criteria:

- Presence of at least one noun/adjective/adverb with a negative connotation (e.g., coldness, insensibility)
- Presence of threats/swear words

We considered only posts and comments which referred to ASL 5 and excluded those which concerned other Local Health Trusts (Tuscany/Emilia Region), inappropriate posts/comments or spam.

We identified and classified the point of view of users who commented and shared posts and developed a thematic matrix through an analysis of the most recurring categories of meaning (framework method). We choose this method because “this approach identifies similarities and differences in qualitative data, before focusing on relationships between different parts of the data, thereby seeking to draw descriptive and/or explanatory conclusions clustered around themes” [23].

The categories of meaning and the themes were evaluated through an inductive approach where “themes are generated from the data though open (unrestricted) coding, followed by refinement of themes.” 

We also contacted the administrator of the largest local Facebook community (S.P.V) to ask about the proportion of offensive posts and comments they deleted during the study period.

## 3. Results

**Figure 2:**
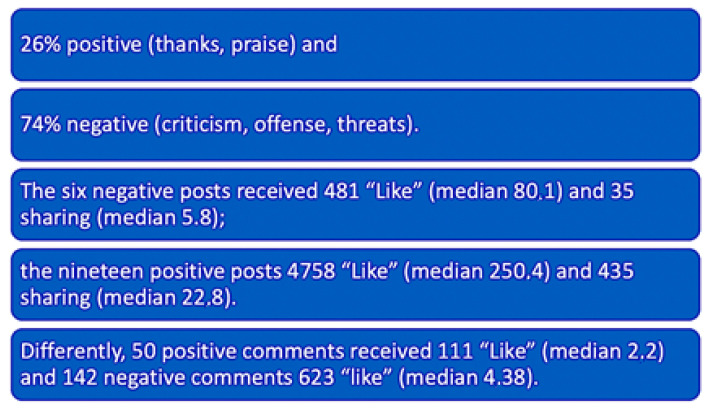
Summary of the main types of comments analyzed in our study.

We collected 217 texts (25 posts and 192 comments): 26% positive (thanks, praise) and 74% negative (criticism, offense, threats). The six negative posts received 481 “Likes” (median 80.1) and 35 shares (median 5.8); the nineteen positive posts received 4758 “Likes” (median 250.4) and 435 shares (median 22.8). Differently, 50 positive comments received 111 “Likes” (median 2.2) and 142 negative comments received 623 “Likes” (median 4.38) (Figure 2).

The analysis highlighted three main meaning categories: Healthcare worker aggressions on social networks included

1 lack of adequate and functional structures
2 negative point of view (POV) of users towards some departments
3 positive POV towards other departments.

Some users understood operators’ difficulties due to adverse working conditions (inadequate and old work environments and staff shortages) and the negative sentiment was fueled by news about the delay in the new hospital construction. Others complained of health providers’ poor humanity and sensitivity (“a course of good manners should be included in the study plan” (referring to the degree course in Nursing); “incapable workers in that hospital ward; lack of skills”). Among the major contributing factors to aggression were the sharing of disparaging news through posts and personal experience of long waiting hours (i.e., in the emergency department), moments in which the negative sentiment leads to the threat of physical aggression (“one should bludgeon them ...” No significant differences were observed between the various categories of healthcare providers. Geriatric, medical wards and emergency departments were the most frequent targets of negative comments. All the posts and comments referred to first-line operators, with just one post for management.

The administrator of the largest local Facebook community, to whom we asked about the number of offensive posts or comments deleted, answered that he could not tell us exactly how many comments were removed, as no trace remains when a comment or post is deleted.

In general, he did not remember many negative or offensive comments addressed to single persons, more often he read complaints about the service, especially for the long waits and for the structures deemed inadequate and dilapidated, and more rarely about misdiagnosis or course with complications. Normally they do not remove negative comments on disservices if they are expressed with decorum and perhaps supported by data or photographs. Reporting a disservice is different from attributing responsibilities (which in their opinion should be searched elsewhere, healthcare workers aggressions not on social networks). If the issue concerns the community, they are inclined to publish (for example, if a department is scrambled, it is scrambled for everyone and it is a fact). If it is a personal process, they believe that the individual experience is not an example.

## 4. Discussion

Incidents of violence by patients against staff are considered a sentinel event that needs systemic analysis [3,24]. To our knowledge, this is the first report on cyberbullying against staff perpetuated by healthcare users, a phenomenon that deserves to be studied because of its potential serious consequences on the psychological and physical health of victims, ranging from anxiety and self-esteem reduction to depression and suicide [25]. In particular, the potential dissemination to an infinite audience and the permanence of digital content are cyber-specific features that contribute to amplifying the harm [22]. There has been a long-standing call for the implementation of a bullying research and verification program, with specific training for health workers to raise awareness of workplace bullying in order to identify cases at an early stage [26,27].

We only focused our research on social networks (Facebook in this case, as it was the most popular in our area), but there are many ways to carry out cyberbullying (e.g., text messaging, email, other social media sites such as Twitter and Instagram, blogs, chat rooms, instant messages, posting photos, videos, etc.) and others are emerging [15]. The published works exploring the role of social networks to capture the patient experience suggest a positive language bias that we did not confirm. Indeed, they include hospitals’ twitter handles, while we focused our attention on local, non-institutional community pages. It is speculated that the institutional character of the accounts included in the study of Hawkins J.B. et al. determined the language bias and explain the surprisingly weak association with one measure of hospital quality (30-day readmission) and no association with Hospital Consumer Assessment of Healthcare Providers and Systems, HCAHPS—an established standard of patient experience [28].

Unfortunately, we have seen a growth of these behaviors during the years, mostly due to the spread of social networks [26]. This phenomenon has been monitored in Italy has had exponential growth, so much so that it is now considered a new social emergency, justifying the promulgation of a law. For example, in the five-year period from 2015–2019, almost 11 thousand cases of aggression against healthcare personnel were estimated by the Italian National Institute Work Accident Insurance (INAIL).

Law no. 113 of 14 August 2020, entitled “Provisions on safety for health and social-health professions in the exercise of their functions,” introduced important innovations into the Italian legal system to curb the phenomenon of verbal and physical violence against all health professionals.

Article 1 immediately defines the recipients of the rule, that is, all those who belong to health professions already recognized and those who will eventually be recognized in the future. To ensure a greater operativeness of the law, Article 2 provides the establishment of a National Observatory on the safety of health and social-health professions at the Ministry of Health. The purpose is to ensure continuous and constant monitoring of the phenomenon of violence against health professionals through:

(a) the facilitation of the process of reporting the act of violence;
(b) the collection of data and their analysis;
(c) the implementation of measures aimed at the prevention of the phenomenon and its repression through a structured system of sanctions [1,6].

In particular, it ensures the monitoring of episodes of violence or sentinel events that may give rise to acts committed with violence or threats to the detriment of health and social-health professions in the exercise of their functions; the monitoring of the implementation of prevention and protection measures to ensure the levels of safety in the workplace, including through the use of video surveillance tools; the promotion of studies and analysis for the formulation of proposals and measures to reduce risk factors in the most exposed environments; the promotion of the dissemination of good practices in the field of safety of health and social care professionals, also in the form of team work; the promotion of training courses for medical and health care personnel, aimed at the prevention and management of conflict situations and to improve the quality of communication with users.

From the analysis of the international scientific literature, it is clear that the risk factors are common, and they are related to the patient, the organization and the professional and all three can contribute to the event of violence. In particular, the following factors contribute most to the onset of violence: disappointed expectations of patients/families, long waiting times, crowding, difficulty with communication and/or collaboration between operators and patients, the presence of a single operator in dislocated and isolated places, lack of staff, the increase of patients with psychiatric disorders, and the spread and abuse of alcohol and drugs [29,30,31].

The introductory report of Italian Law no. 113 confirms this reality, stating that the risk factors responsible for acts of violence directed against health professionals are numerous, but emphasizing that the peculiar and recurring element is represented by the highly interactive and personal relationship that is established between the patient and the health care provider during the provision of health care services. This often involves subjects, such as the patient himself or family members, who are in a state of vulnerability, frustration or loss of control, especially if under the influence of alcohol or drugs.

It is important to emphasize how the onset of this phenomenon is further stimulated by the current pandemic context of COVID-19 [32,33].

The World Health Assembly COVID-19 pandemic response suggests:

(1) Adequate accountability mechanism by governments against perpetrators of violence against health personnel;
(2) Accurate and systematic data collection on violent incidents in relation to the pandemic to document the phenomenon across the globe;
(3) Effective information campaigns to keep the public informed and stop misinformation related to COVID-19 contamination;
(4) Close cooperation between local/state authorities, health professionals’ organizations and other relevant health actors, as well as media organizations, to denounce and prevent the problem of violence [34].

We started our work searching on the Medline, Embase and Scopus database: we found many articles about non-physical violence, and some healthcare companies have been monitoring violence on health providers for many years, but most of these articles were referring to the period from 2011 to 2014, when the age of Facebook users was lower than today. Furthermore, the number of Italian registered users grew from 20 to 29 million in ten years. In consideration of such scarce literature, we looked at the real world and carried out a pilot qual- and quantitative study searching Facebook pages, groups, posts and comments relating to the Local Health Trust (ASL 5 La Spezia, Liguria), using keywords like “Nurses,” “Doctor,” “Assault” and “Hospital.” The oldest retrieved post dated back to 2013. We analyzed four Facebook pages (with a total of 190,000 followers, a very representative sample considering that ASL5 serves a population of about 220,000 individuals), four main local Facebook groups (about 41,000 followers) and one website of hospital reviews by patients. We collected 217 texts (25 posts and 192 comments): 26% positive (thanks, praise) and 74% negative (criticism, offense, threats).

Negative comments received about double the “Likes” than the positive ones. During the COVID-19 emergency, posts supporting health providers increased, but this trend lasted only a few months.

Some users understand operators’ difficulties due to adverse working conditions (inadequate and old work environments, staff shortage) and the negative sentiments are fueled by news about the delay in the new hospital construction. Some others complained of health providers’ poor humanity and sensitivity. No significant differences were observed between the various categories of healthcare providers. Geriatric, medical wards and emergency departments were the most frequent target of negative comments. All the posts and comments referred to first-line operators, with just one to management.

Our findings highlight there is a clear “distance” between healthcare users and operators, generated by lack of information on the one hand and poor working conditions affecting communication on the other. Moreover, there are contributing factors like pressure, long waits, discomfort, etc., that need to be identified and addressed. Compared to traditional face-to-face bullying, cyberbullying aggressions towards healthcare workers on social networks can be more dangerous as it is contagious and diffusive, overcoming spatial, temporal and personal boundaries.

Online there is the tendency for individuals to say or behave in a manner that would not be used during face-to-face interactions, as if “they do not have a filter for their communication” [22].

Although this study has some limits, such as a restricted field of observation (only local Facebook pages and groups), non-exhaustiveness due to the censorship applied by the administrators of the page to some posts, and the lack of an impact analysis of such assaults on victims, we hope it will have the merit of drawing attention to the problem [35,36,37]. At present, we are not aware of desperate actions by cyber-bullied healthcare providers, but a few weeks ago in Italy a policeman died by suicide because he was targeted by insults on social networks.

As “not all evil comes to harm,” we also believe that healthcare companies should monitor social networks constantly to anticipate eventual censorship and critically analyze posts and comments in order to learn from user experiences and improve organizations and behaviors, if needed.

## 5. Conclusions

In conclusion, we agree that our study is small and that a larger study is necessary, but we would like to shine a light on this emerging phenomenon because of its potentially serious consequences.

Compared to traditional face-to-face bullying, cyberbullying can be more dangerous as it is contagious and diffusive, without spatial, temporal or personal boundaries.

The internet has provided us with a lot of advantageous possibilities in terms of information, education, games and social interactions [38,39]. However, connecting people in real time and from anywhere has also disclosed negative implications. Cyberbullying is among them.

Our findings show that online violence against healthcare providers is a real, even if still unknown, problem that needs immediate and concrete caution. Healthcare worker aggression on social networks is a real problem that is expanding and is complicated to repress because it is complicated to detect due to the extent of the public domain. From this point of view, local community pages rather than hospitals’ accounts can provide hospital administrators, policymakers, physicians and researchers with untapped and less filtered information on quality of care. On the other hand, health support should be offered to the victims and legal measures taken against the authors of offensive, threatening or discrediting posts to protect healthcare workers and deter further episodes.

## Data Availability

Not applicable.
